# Supplementary material for: Self-Reported Purchasing Behaviour, Sociodemographic Predictors of Plant-Based Protein Purchasing and Knowledge about Protein in Scotland and England
Source: Nutrients. 2022 Nov 7;14(21):4706. doi: 10.3390/nu14214706 (PMC9657987; doi:10.3390/nu14214706)
Supplement: Supplementary file 1 [file nutrients-14-04706-s001.zip › nutrients-1979324-supplementary.pdf]

**S1.** Survey Questions.

**What Plants are on your Plate?**

1. Please read here Participant Information Sheet for a brief summary of what you will be asked to do. When you proceed to the next page, please read each statement carefully and click on the Agree button if you give consent.

---

2. I have read and downloaded a copy of the Participant Information Sheet to keep and agree to take part in this study.

   I understand that my participation in this project will involve the completion of a short online survey that will take around 15-20 minutes of my time.

   I understand that participation in this study is entirely voluntary and that I can withdraw from the study at any time without providing a reason by simply closing the browser. I am aware that this may lead to loss of some/all incentive offered by the research panel.
   - ○ Agree
   - ○ Disagree

---

3. I understand that the information provided by me will be held anonymously so that it is impossible to trace this information back to me individually. Information will be held on a secure server and will be retained for 10 years.

   I understand that, because data collection is anonymous, I will be unable to withdraw after submitting my data as it will not be possible to identify me within the dataset.

   To ensure access to the data for the wider research community, I understand the anonymous dataset may be archived in an online repository (e.g., the Open Science Framework, https://osf.io/) or sent to other researchers upon request for inspection.
   - ○ Agree
   - ○ Disagree

---

4. I confirm that I am 18 years or older, currently living in the United Kingdom, and that I have a good understanding of the English language.
   I confirm that the purpose, participation information, and data storage of this research have been sufficiently explained.
   - ○ Agree
   - ○ Disagree

*Skips to: End of Survey if Disagree is selected for Questions 2, 3 or 4*

**End of Block: PIS and Consent**

---

**Start of Block: Captcha**

5. Before you proceed to the survey, please tick the Captcha below.

**End of Block: Captcha**

---

Start of Block: Quota questions

6.

6a. Which country do you live in?

- ◯ Scotland
- ◯ England
- ◯ Wales
- ◯ Northern Ireland
- ◯ None of the above

*Skips to: End of Survey if Wales, Northern Ireland or None of the above is selected*

6b. What is your postcode? (please note this will be coded to maintain your anonymity)

- ◯ Postcode ______

*(IMD level and urban/rural classification will be identified from postcode)*

End of Block: Quota questions

---

Start of Block: Demographics

**Section 1 - Background Questions**

7. How old are you?

*(Dropdown list of ages from 18-100)* ______

8. What is your gender?

- ◯ Male
- ◯ Female
- ◯ Non-binary
- ◯ Prefer not to say

9. What is your ethnicity?

- ◯ White
- ◯ Black/African/Caribbean
- ◯ Asian
- ◯ Arab
- ◯ Mixed/Multiple Ethnic groups
- ◯ Other, please specify________________________________________________
- ◯ Prefer not to answer

10.

10a. What is your height? Please select your preferred option

- ◯ in feet and inches
- ◯ in metres

10b. What is your height? (if feet and inches is selected in Q9a)

(Dropdown lists from 4-6ft and 0-11inches)_____

9b. What is your height? (if metres is selected in Q9a)
*(Dropdown list from 1.20-2.10m)*_____

11.

11a. What is your weight? Please select your preferred option.

- ○ in stones and pounds
- ○ in kilograms

11b. What is your weight? (if stones and pounds is selected in Q10a)
*(Dropdown lists from 4-30stone and 0-13pounds)* ______

10b. What is your weight? (if kilograms is selected in Q10a)
*(Dropdown list from 25-200kg)* ______

12. What is your household's total income from all sources over the last 12 months, before tax and other deductions?
Each response option below includes equivalent weekly income (left) and yearly income (right).
If you are unsure, please provide your best estimate.

- ○ Less than £100 / Less than £5,200
- ○ £100 to £199 / £5,200 to £10,399
- ○ £200 to £299 / £10,400 to £15,599
- ○ £300 to £399 / £15,600 to £20,799
- ○ £400 to £499 / £20,800 to £25,999
- ○ £500 to £699 / £26,000 to £36,399
- ○ £700 to £999 / £36,400 to £51,999
- ○ £1000 to £1499 / £52,000 to £77,999
- ○ £1,500 or more / £78,000 or more

13. How many people live in your household?

- Adults (over 16 years)___________
- Children (under 16 years)________

*(Dropdown lists from 0-10 for adults and children)*

14. Do you eat a prescribed diet for a medical condition?

- ○ Yes, e.g. for diabetes, coeliac, other (please specify) ____________________
- ○ No

15. Do you follow any other specific diet?

- ○ Yes, Vegetarian
- ○ Yes, Vegan
- ○ Yes, Other, please specify ______________________________
- ○ No

**End of Block: Demographics**

---

**Start of Block: General Food Shopping**

**Section 2 - General Food Shopping**

16. Where do you do most of your food shopping?
    - ◯ In store
    - ◯ Online
    - ◯ Food bank

17. At which supermarket do you do most of your food shopping?
    (either in store or online, please select **one** option)
    - ◯ Aldi
    - ◯ Asda
    - ◯ Budgens
    - ◯ The Co-operative
    - ◯ Dunnes Stores
    - ◯ Farmfoods
    - ◯ Iceland
    - ◯ Lidl
    - ◯ Marks & Spencer
    - ◯ Morrisons
    - ◯ Ocado (online shopping)
    - ◯ Sainsburys
    - ◯ Scotmid Co-operative
    - ◯ SuperValu
    - ◯ Tesco
    - ◯ Waitrose
    - ◯ Other, please specify ________________________________

---

18. How much do you spend on your average weekly food shop?
    - ◯ less than £30
    - ◯ £30-£40
    - ◯ £40-£50
    - ◯ £50-£60
    - ◯ £70-£80
    - ◯ £90-£100
    - ◯ more than £100

19. What is the most important influence on your food shopping choices?
Thinking about individual items you buy, please rank in order the top **five** most important considerations for you, (1 = most important, 5 = least important)
*(list is randomised in survey)*

______ Price
______ Brand Loyalty
______ Healthy Option
______ Promotions or Offers
______ Eco-friendly Option
______ Organic Option
______ Use or Sell by Dates
______ Familiarity
______ Ease of Use
______ Reduced Cost Brand
______ Freshness
______ Quality
_______Taste preference

End of Block: General Food Shopping

---

Start of Block: Consumption of Specific Foods

**Section 3 - Consumption of Specific Foods**

*20.* Do you buy, or prepare, any of these food products, at least once a week?

Please select **all** that apply *(list is randomised in survey)*

☐ Chicken and Turkey dishes
☐ Beef and Veal dishes
☐ Baked Beans
☐ Eggs
☐ Bacon and Ham
☐ Meat Pies and Pastries
☐ Oily Fish (herring, mackerel, tuna, salmon)
☐ Sausages
☐ Pork and Pork dishes
☐ White Fish (cod, haddock)
☐ Cheese, Milks and Yoghurts (Dairy or Non-dairy)

21.

21a. Do you buy the following plant-based foods?

| | Yes | No |
|---|---|---|
| Green Beans or Peas | ○ | ○ |
| Beans | ○ | ○ |
| Lentils | ○ | ○ |
| Quorn™ Products | ○ | ○ |
| Soya Products | ○ | ○ |
| Other Meat Free Products | ○ | ○ |

You will now be asked more specific questions about the foods you answered 'Yes' to.

*Questions 21b-g only displayed if the answer to Question 21a was 'Yes'*

21b. Which types of **Green Beans** or **Peas** do you buy?
Please select **all** that apply

| | Fresh | Frozen | Canned |
|---|---|---|---|
| Green Beans | ☐ | ☐ | ☐ |
| Garden Peas | ☐ | ☐ | ☐ |
| Petit Pois | ☐ | ☐ | ☐ |
| Chickpeas | ☐ | ☐ | ☐ |
| Black Eyed Peas | ☐ | ☐ | ☐ |
| Other | ☐ | ☐ | ☐ |

21c. Which types of **Beans** do you buy?
Please select **all** that apply

| | Fresh | Frozen | Canned | Dried |
|---|---|---|---|---|
| Baked Beans | ○ | ○ | ○ | ○ |
| Kidney Beans | ○ | ○ | ○ | ○ |
| Broad Beans (Fava) | ○ | ○ | ○ | ○ |
| Borlotti Beans | ○ | ○ | ○ | ○ |
| Other | ○ | ○ | ○ | ○ |

21d. Which type of **Lentils** do you buy?

- ☐ Dried
- ☐ Pre-prepared

21e. Which types of **Quorn™ Products** do you buy?

Please select **all** that apply *(list is randomised in survey)*

- ☐ Fillets or Pieces
- ☐ Breaded or Coated
- ☐ Mince or Meatballs
- ☐ Burgers or Sausages
- ☐ Pies or Pastries
- ☐ Other, please specify________________________________

21f. Which types of **Soya Products** do you buy?
Please select **all** that apply *(list is randomised in survey)*

- ☐ Soya Beans
- ☐ Textured Vegetable Protein (TVP)
- ☐ Tofu
- ☐ Soya Meat Substitute
- ☐ Soya Milk
- ☐ Soya Yoghurt
- ☐ Other, please specify________________________________

21g. Which types of **Other Meat Free Products** do you buy?
Please select **all** that apply *(list is randomised in survey)*

- ☐ Nut Roast
- ☐ Fillets or Pieces
- ☐ Breaded or Coated
- ☐ Mince or Meatballs
- ☐ Burgers or Sausages
- ☐ Pies or Pastries
- ☐ Dairy Free Cheese
- ☐ Other, please specify____________________________________

22. For what reasons may you find it difficult to consume plant-based meals in your diet?
e.g. meals with beans, peas, lentils or meat substitutes
Please select **all** that apply *(list is randomised in survey)*

- ○ I don't know how to cook them
- ○ I don't like the taste of them
- ○ I prefer meat-based meals
- ○ It's too time consuming to prepare meals with them
- ○ I don't like cooking my own meals
- ○ There's not enough choice in the supermarket
- ○ My budget is limited so I buy foods I am familiar with
- ○ Other, please specify ____________________________________

23. On average, how many portions of fruit and vegetables do you consume each day?
*Do not count potatoes, yams, cassava or plantain*
*(Dropdown list from 0-10)*__________

Please see some examples below from the NHS website of what one portion of fruit and vegetables is:

| **FRUIT** | **VEGETABLES** |
|---|---|
| Small fresh fruit<br>(2 or more small fruit)<br>• 2 plums<br>• 2 satsumas<br>• 2 kiwi fruit<br>• 3 apricots<br>• 6 lychees<br>• 7 strawberries<br>• 14 cherries | Green vegetables<br>• 2 spears of broccoli<br>• 4 heaped tbsp of cooked kale, spinach, spring greens or green beans |
| Medium fresh fruit<br>(1 medium fruit)<br>• apple, banana, pear, orange or nectarine | Cooked vegetables<br>(fresh, frozen or canned)<br>• 3 heaped tbsp of carrots, peas or sweetcorn<br>• 8 florets of cauliflower |
| Large fresh fruit | Salad vegetables |

| | |
|---|---|
| • half a grapefruit<br>• 1 slice of papaya<br>• 1 slice of melon (5cm slice)<br>• 1 large slice of pineapple<br>• 2 slices of mango (5cm slices) | • 1.5 full-length sticks of celery<br>• a 5cm piece of cucumber<br>• 1 medium tomato<br>• 7 cherry tomatoes |
| Dried fruit (around 30g)<br>• 1 heaped tablespoon of raisins, currants or sultanas<br>• 2 figs<br>• 3 prunes<br>• 1 handful of dried banana chips | |
| Tinned or canned fruit<br>(similar to fresh fruit)<br>• 2 pear or peach halves<br>• 6 apricot halves<br>• 8 segments of grapefruit | |
| Fruit juices, vegetable juices or smoothies<br>• 1 or more glasses daily only ever counts as 1 portion | |

24. Where do you buy meat from?

○ Supermarket
○ Butcher
○ Local market
○ Other, please specify________________________________________________
○ I don't buy meat

25.

25a. Have you tried to reduce your red meat purchases (e.g. beef, pork, lamb) in the last 6months?

○ Yes
○ No

25b. If YES, why?

Please select **one** answer *(list is randomised in survey)*

○ Health
○ Animal welfare
○ Finance
○ To reduce carbon footprint
○ Other, please specify________________________________________

End of Block: Consumption of Specific Foods

Start of Block: Protein

**Section 4 - Protein**

26. If a food is labelled 'high in protein' do you think it means: *(list is randomised in survey)*

○ It is healthy
○ It is unhealthy

*(list is randomised in survey)*

27. Do you look for food products that are labelled 'high in protein'?
   - ○ Yes
   - ○ No

28. Please rate how you feel about the following **seven** statements about **PROTEIN** *(list is randomised in survey)*

| | Agree | Neither agree nor disagree | Disagree |
|---|---|---|---|
| Protein is important for a healthy body | ○ | ○ | ○ |
| Protein is important for healthy body weight | ○ | ○ | ○ |
| Protein is important for healthy bones | ○ | ○ | ○ |
| Protein is important for body muscle | ○ | ○ | ○ |
| Protein is important for healthy cholesterol levels (blood fat) | ○ | ○ | ○ |
| Protein is important for healthy glucose levels (blood sugar) | ○ | ○ | ○ |
| Protein is important for healthy blood pressure | ○ | ○ | ○ |

29. Please rate how you feel about the following **six** statements about **PROTEIN SOURCES** *(list is randomised in survey)*

| | Agree | Neither agree nor disagree | Disagree |
|---|---|---|---|
| Mushrooms are a good source of protein | ○ | ○ | ○ |
| Peanuts are a good source of protein | ○ | ○ | ○ |
| Lentils are a good source of protein | ○ | ○ | ○ |
| Potatoes are a good source of protein | ○ | ○ | ○ |
| Oats are a good source of protein | ○ | ○ | ○ |
| Rice is a good source of protein | ○ | ○ | ○ |

30. Please rate how you feel about the following **six** statements about **EATING SPECIFIC DIETS** *(list is randomised in survey)*

| | Agree | Neither agree nor disagree | Disagree |
|---|---|---|---|
| Eating too much protein is bad for your gut (gastro-intestinal tract) | ○ | ○ | ○ |
| Eating a plant-based diet (e.g. vegetarian or vegan) can lower the risk of developing Type 2 Diabetes (elevated blood sugar) | ○ | ○ | ○ |
| Eating less meat is good for the planet | ○ | ○ | ○ |
| Eating a plant-based diet (e.g. vegetarian or vegan) is good for the planet | ○ | ○ | ○ |
| Eating a more plant-based diet is healthy | ○ | ○ | ○ |
| Eating a diet to include red meat (beef, pork, lamb) is healthy | ○ | ○ | ○ |

**End of Block: Protein**

**End of Survey**

We thank you for your time spent taking this survey.

Your response has been recorded.

To find out more about the research we do at Aberdeen University please visit our website (see link below)

[Aberdeen University Research]
